# Supplementary material for: Association between oral microbiome and seven types of cancers in East Asian population: a two-sample Mendelian randomization analysis
Source: Front Mol Biosci. 2023 Nov 21;10:1327893. doi: 10.3389/fmolb.2023.1327893 (PMC10702768; doi:10.3389/fmolb.2023.1327893)
Supplement: Supplementary file 1 [file DataSheet1.ZIP › Supplementary Materials/MR plots of saliva/saliva═╝/ovarian cancer/pheno.743_to_ovarian cancer_forest.pdf]

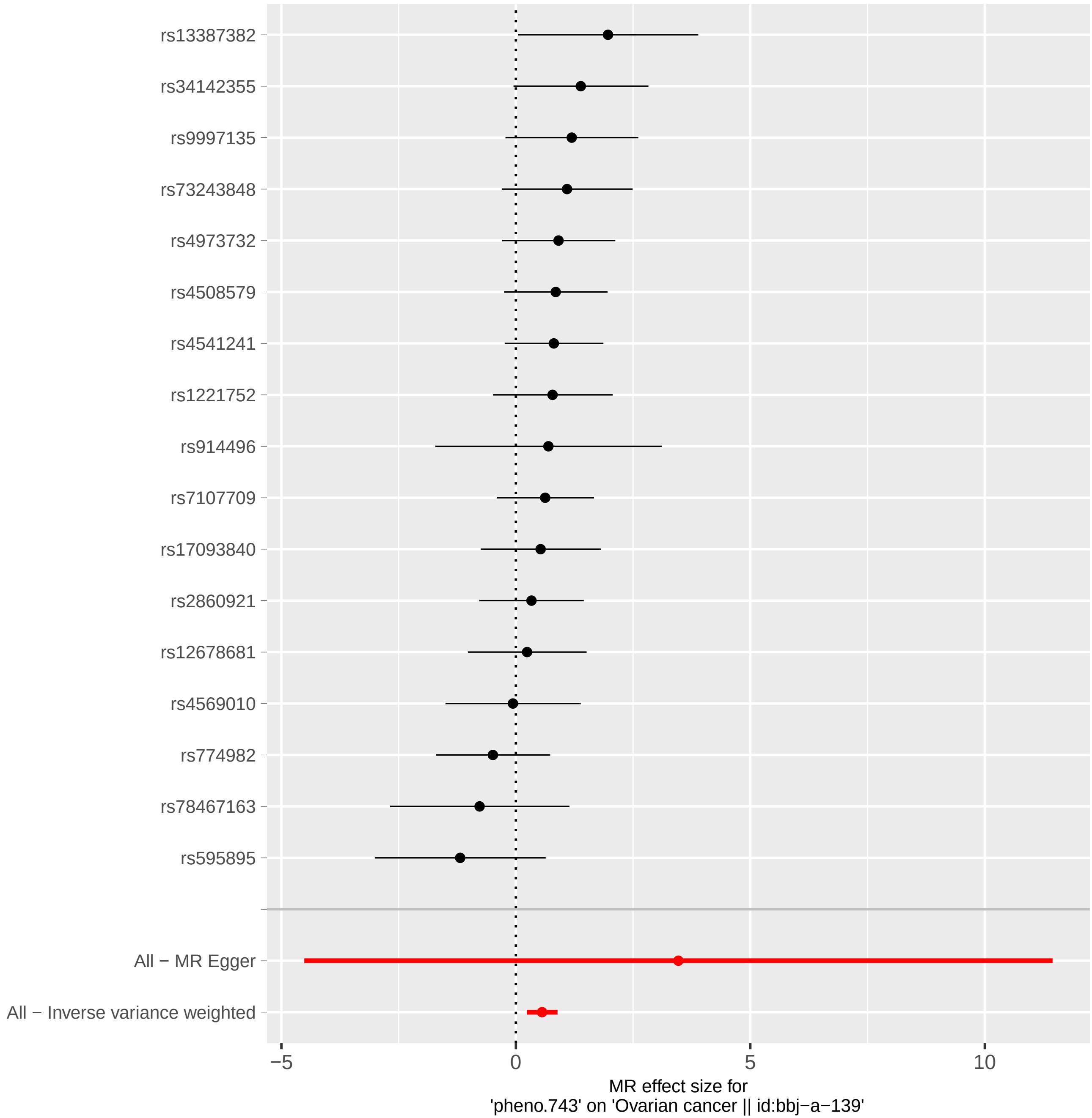
rs13387382
rs34142355
rs9997135
rs73243848
rs4973732
rs4508579
rs4541241
rs1221752
rs914496
rs7107709
rs17093840
rs2860921
rs12678681
rs4569010
rs774982
rs78467163
rs595895
All − MR Egger
All − Inverse variance weighted
−5
0
5
10
MR effect size for
'pheno.743' on 'Ovarian cancer || id:bbj−a−139'
